# Camel milk concerning erythrocytic immune-related gene expression in *Escherichia coli*-challenged broilers

**DOI:** 10.3389/fvets.2026.1960495

**Published:** 2026-10-15

**Authors:** Uroosa Arain, Nasir Rajput, Mahum Arain, Ali Raza Jahejo, Yunkyoung Lee, Guiguo Zhang

**Affiliations:** 1 Department of Animal Nutrition, Shandong Agricultural University, Taian, China; 2 Department of Poultry Husbandry, Sindh Agriculture University, Tandojam, Pakistan; 3 Department of Food Science and Nutrition, Jeju National University, Jeju, Republic of Korea

**Keywords:** camel milk, erythrocyte, *Escherichia coli*, immunity, poultry, production

## Abstract

**Background:**

Chicken erythrocytes play a role in immunity, yet the function of erythrocyte-related immune genes during *Escherichia coli* (*E. coli*) infection remains underexplored. This study investigated the effects of camel milk powder supplementation on growth performance and erythrocyte-specific immune gene expression in *E. coli*-challenged broiler chickens.

**Methods:**

A total of 240 one-day-old broiler chickens were randomly assigned to four groups with three replicates (20 Chickens per replicate): Control (Basal diet + PBS), Infected (*E. coli* challenged), T1 (*E. coli* + 5 g/kg camel milk), and T2 (*E. coli* + 10 g/kg camel milk). Performance metrics, organ weights, intestinal and hepatic histomorphology, hematological parameters, and viable *E. coli* counts in blood and cecum were assessed. Bioinformatics analysis of microarray data (GPL8764) guided the selection of key immune genes. Quantitative real-time PCR was performed on purified erythrocytes to evaluate immune gene expression.

**Results:**

Feed intake, weight gain, and FCR were significantly (*p* < 0.05) improved in the T1 group compared to the Infected group.While relative weights of the spleen and bursa did not differ significantly among groups, liver weight was influenced by supplementation, and histomorphological analysis revealed improved intestinal and hepatic architecture in camel milk-supplemented chickens. Hematological parameters (RBC, WBC, PCV, Hb) disrupted by infection were restored in treated groups. Furthermore, viable E. coli counts in the blood and cecum were significantly reduced in T1 and T2 groups on days 28 and 35. Bioinformatics analysis of microarray data (GPL8764) guided the selection of key immune genes. Quantitative real-time PCR performed on purified erythrocytes demonstrated that camel milk supplementation modulated the expression of Interleukin genes (IL-2, IL-6, IL-10, and IL-12) in Infected chickens.

**Conclusion:**

The camel milk supplementation at 5 g/kg, effectively mitigated the negative impacts of *E. coli* infection by enhancing growth performance, reducing bacterial load, and modulating immune responses.

## 1 Introduction

Global demand for poultry products (meat and eggs) is rising rapidly, with total meat consumption estimated to increase to approximately 45.3 kg per capita by 2030 (1). However, optimizing production and performance is heavily dependent on controlling infectious diseases, including Avian colibacillosis caused by *Escherichia coli* (*E. coli*) (2). This disease causes severe health complications and significant financial losses in the poultry industry. Although *E. coli* is a natural inhabitant of the chicken intestine, it acts as an opportunistic pathogen capable of causing disease under conditions of stress, overpopulation, poor ventilation, starvation, temperature extremes, or immunosuppression (2).

Historically, antibiotics have been extensively used to prevent and treat colibacillosis. However, collective reports indicate a sharp rise in drug resistance among *E. coli* strains, creating a major hurdle in clinical veterinary medicine (3–6). While antibiotics effectively combat pathogens, their broad-spectrum activity often leads to the unintended destruction of beneficial gut commensals. Consequently, urgent interventions are required to restrain the emergence of antimicrobial resistance, leading regulatory bodies like the European Union to restrict specific dietary antibiotics as growth promoters (7). Therefore, identifying safe, antibiotic-free feed additives—such as herbal extracts, organic acids, and probiotics—has become a priority for the poultry sector (8).

Camel milk is recognized for its unique bioactive compounds and has been used traditionally to treat various ailments due to its anti-diabetic, antibacterial, and anti-hepatitis properties (9–11). It contains high levels of lactoferrin, lysozyme, immunoglobulins, and free oleic acids, which resist bacterial contamination (12). Specifically, lactoferrin, lysozyme, and lactoperoxidase in camel milk exhibit germicidal activity against both Gram-positive and Gram-negative microorganisms, including *Staphylococcus aureus*, *Listeria monocytogenes*, and *E. coli*. Lactoferrin is particularly notable as a heme-binding protein; by sequestering iron, it not only inhibits bacterial growth but also prevents oxidative damage to erythrocytes, thereby maintaining their integrity and function (13–15).

Unlike mammalian red blood cells, chicken erythrocytes are nucleated and contribute significantly to immune responses (16). Recent research indicates that these cells participate directly in immunity by responding to pathogens and pro-inflammatory stimuli (13, 14, 17–19). Chicken erythrocytes express Toll-like receptors (TLRs 2, 3, 4, 5, and 7) and are capable of initiating cytokine transcription and releasing heat-sensitive molecules (16). These actions can boost the antiviral activity of macrophages and lead to enhanced production of type I interferons (IFNs) and interleukins (e.g., IL-8) (20–22).

Despite the known antibacterial qualities of camel milk, its regulatory effect on host immune gene expression—particularly within chicken erythrocytes—during an *E. coli* challenge remains underexplored (23). This study integrates transcriptomic profiling of chicken erythrocytes with dietary intervention to offer novel insights into non-antibiotic strategies for poultry health. Therefore, this study was designed to evaluate the protective effects of camel milk powder supplementation on the growth performance and physiological health of broiler chickens challenged with *E. coli*. Furthermore, given the unique immunological role of nucleated avian blood cells, the study specifically aimed to investigate the regulatory influence of camel milk on immune-related gene expression within erythrocytes, providing a comprehensive understanding of its potential as a non-antibiotic feed additive.

## 2 Materials and methods

### 2.1 Experimental design

The study was conducted at Shandong Agricultural University, Taian, China, to determine the effect of camel milk supplementation in the feed of broiler chickens challenged with *E. coli*, a completely randomized design. A total of 240 one-day-old Cobb broiler chickens were reared for 35 days in an environmentally controlled house. The chickens were kept in floor pens ( 1.5×1.5 m) with fresh wood shavings as bedding. The brooding temperature was maintained at $33^{\circ }C$ during the first week and gradually reduced to $24^{\circ }C$ by the end of the experiment, with relative humidity maintained between 60% and 65% . A continuous lighting program ( 23 hours light: 1 hour dark) was provided using white LED bulbs. The facility utilized a mechanical ventilation system to ensure air quality. Feed was provided via manual tube feeders, and fresh tap water was available *ad libitum* through nipple drinkers. The chickens were fed a starter diet from days 1 to 21 and a finisher diet from days 22 to 35. Strict biosecurity measures were implemented, including insect and rodent-proofing, and the facility was cleaned and disinfected with Virkon-S (DuPont, Sudbury, UK) prior to the chickens’ arrival; Infected and Control groups were housed in separate rooms with dedicated personnel and protective clothing to prevent cross-contamination. All chickens were vaccinated against Newcastle Disease and Infectious Bronchitis on day 7, and no antibiotics or coccidiostats were administered in the feed or water throughout the trial. Clinical health and mortality were assessed twice daily by monitoring for signs such as lethargy, diarrhea, and ruffled feathers; any deceased chickens were weighed and recorded to adjust performance data.

Camel milk powder supplementation began on day 1 and continued until day 35, while *E. coli* infection was induced on the 7th day. The chickens were divided into four groups consisting of three biological replicates per group with 20 chickens per replicate ( n=60 chickens per group; Total N=240 ): a Control group receiving the basal diet and a sterile PBS mock challenge (no infection, no milk), an Infected group receiving the basal diet and the *E. coli* O78 challenge (no milk), T1 (*E. coli* + 5 g/kg camel milk), and T2 (*E. coli* + 10 g/kg camel milk) (Table 1). Infected and Control groups were housed in separate rooms with dedicated personnel and protective clothing to prevent cross-contamination.

**Table 1:** Experimental design, treatment groups, and chronological sampling schedule.

Experimental design				
Group name	Control	Infected	T1	T2
Treatment description	Basal diet + PBS mock challenge	Basal diet + *E. coli* challenge	Basal diet + *E. coli* + 5 g/kg Camel Milk	Basal diet + *E. coli* + 10 g/kg Camel Milk
Replicates	3	3	3	3
Chicken per replicate	20	20	20	20
Total chickens ( N )	60	60	60	60
Total study population	240 Broiler Chickens			

Timeline and sampling schedule				
Time point	Event/Activity	Samples collected	Target analysis	Sample size ( n )
Day 1	Start of experiment	—	Start of Camel Milk supplementation (T1, T2)	—
Day 7	Infection challenge	—	Intranasal *E. coli* O78 induction (Infected, T1, T2)	—
Day 14	Sampling	Blood (EDTA)	*E. coli* enumeration	2 chickens/rep ( n=6 /group)
		Cecal Digesta	*E. coli* enumeration	2 chickens/rep ( n=6 /group)
		Blood (Whole)	RNA extraction (Gene Expression)	1 chicken/rep ( n=3 /group)
Day 21	Sampling	Blood (EDTA)	*E. coli* enumeration	2 chickens/rep ( n=6 /group)
		Cecal Digesta	*E. coli* enumeration	2 chickens/rep ( n=6 /group)
Day 35	Final Sampling & Slaughter	Blood (EDTA)	Hematology & *E. coli* enumeration	2 chickens/rep ( n=6 /group)
		Cecal Digesta	*E. coli* enumeration	2 chickens/rep ( n=6 /group)
		Liver, Spleen, Bursa	Relative organ weights	2 chickens/rep ( n=6 /group)
		Liver & Duodenum Tissue	Histomorphology	4 chickens/group ( randomly selected fromn=6 )

### 2.2 Camel milk

The freeze-dried camel milk powder used in this study was purchased online from DNS Global Foods (Manjusar GIDC, Savli, Vadodara, Gujarat, India). The product was prepared from pasteurized fresh camel milk prior to freeze-drying and contained the following nutritional values per 100 g: energy 453.39 Kcal, carbohydrate 50.16 g, protein 21.90 g, total fat 18.35 g, calcium 720.14 mg, vitamin C 12.80 mg, and iron 0.55 mg.

The basal diet was formulated as a mash (Table 2) following a regional rice-fishmeal based formulation commonly used in poultry research in China and Pakistan (24). While the metabolizable energy values were slightly lower than the global Cobb 500 recommendations, this formulation provided a consistent baseline to evaluate the comparative effects of the supplements across all experimental groups. To ensure the diet was nutritionally complete, a comprehensive vitamin and mineral premix was supplemented at a rate of 5 g/kg, and salt was added to meet the requirements of the Cobb 500 broiler strain. For the treatment groups (T1 and T2), the camel milk powder was manually mixed into the basal diet daily to ensure uniform distribution; the diets were not pelleted to preserve the bioactivity of the milk components. Due to the low inclusion rates (0.5 and 1.0%), the proximate composition (Crude Protein and Metabolizable Energy) of the supplemented diets remained comparable to the basal diet values listed in Table 2. The feed contained no added antimicrobials or coccidiostats.

**Table 2:** Composition of basal diet for broiler chickens and nutritive values.

Ingredients	Starter feed (g/kg)	Finisher feed (g/kg)
Rice	316	400
Maize	100	100
Rice polish	150	160
Fish meal	85	80
Soya bean	70	55
Guar meal	50	40
Canola meal	115	80
Rape seed meal	33	30
Sunflower	70	44
Lime stone	11	11
Nutritive values of experimental diet		
Crude protein	21.2	19
Metabolize energy	2,800 K/Cal	2,950 K/Cal

Mineral and vitamin premix was supplemented at 5 g/kg of diet, containing: transretinyl acetate (25 mg), cholecalciferol (6 mg), menadione (1.2 mg), thiamine (2.3 mg), riboflavin (8 mg), nicotinamide (42 mg), choline chloride (400 mg), calcium pantothenate (10 mg), pyridoxine HCl (4 mg), biotin (0.04 mg), folic acid (1 mg), Cobalamin (0.012 mg), Fe (82 mg), Cu (7.5 mg), Mn (110 mg), Zn (64 mg), I (1.1 mg), and Se (0.28 mg). Salt was added as required to meet nutritional needs.

### 2.3 *Escherichia coli* dose preparation and induction

To prepare the *E. coli* O78 (CVCC1555) inoculum, the pathogenic strain was first incubated aerobically for 24 h at 37 °C with 120 rpm shaking in Luria-Bacterial liquid medium (Solarbio Life Sciences, Beijing, China). Following incubation, the bacterial culture was centrifuged at 2800 g for 10 min and subsequently washed three times with phosphate-buffered saline (PBS) (Thermo Fisher Scientific, Waltham, MA, USA). A spectrometer at 600 nm was used to measure the bacterial concentration, which was confirmed by plating serial dilutions on agar plates. We then diluted the suspension with PBS to achieve a target concentration of 10^4 colony-forming units (CFU)/ml in a 0.5 mL volume, suitable for inducing *E. coli* infection (25). The infection was administered intranasally, a procedure detailed by Peighambari, et al. (26). The intranasal route was selected to mimic the natural respiratory entry of APEC O78 and to induce systemic colibacillosis. This model allows for the evaluation of bacterial translocation from the respiratory tract into the bloodstream and subsequent dissemination to visceral organs, specifically the liver and intestines.

### 2.4 Performance, immunity and lymphoid organs

Body weight (BW) and daily feed intake (DFI) were recorded for each pen throughout the experiment (days 1 to 35) to calculate the Feed Conversion Ratio (FCR) (27). On day 35, two chicken per replicate ( n=6 per group), were randomly selected and euthanized. The spleen, bursa of Fabricius, and liver were harvested and weighed to determine relative organ weights (organ weight/body weight × 100). Additionally, tissue samples from the liver and duodenum were collected for histomorphological analysis.

### 2.5 Enumeration of *Escherichia coli* in blood and Cecal contents

To monitor the bacterial load, samples were collected on days 14, 21, and 35. Two chicken per replicate ( n=6, per group) were randomly selected.

Blood samples were collected aseptically from the wing vein into sterile tubes containing sodium citrate. Samples were mixed with brain–heart infusion broth (Oxoid Ltd., Basingstoke, UK) at a ratio of 1 part blood to 2 parts broth (3-fold dilution), followed by tenfold serial dilutions. Aliquots (0.1 mL) were spread in duplicate onto MacConkey agar plates and incubated at 37 °C for 24 h. The limit of detection (LOD) for viable *E. coli* in the original blood samples was determined to be 30 CFU/mL.

One gram of cecal digesta was collected from the same chickens and homogenized in 9 mL of sterile nutrient broth. Ten-fold serial dilutions were prepared, and 0.1 mL of each dilution was spread onto MacConkey agar plates incubated at 37 °C for 24 h.

Viable *E. coli* colonies were counted, and results were expressed as colony-forming units (CFU) per mL of blood or per gram of cecal content.

### 2.6 Haematology

On day 35, blood samples (2 mL) were collected from the wing vein of two chickens per replicate ( n=6 per group) into tubes containing EDTA. These samples were processed immediately to quantify red blood cells (RBC), white blood cells (WBC), hemoglobin (Hb), and packed cell volume (PCV), following standard protocols outlined by Jahejo et al. (27).

### 2.7 Intestinal microflora

Initially, bacterial associates were enriched by culturing them in Nutrient broth media. *Escherichia coli* was then specifically cultured on MacConkey agar plates under aerobic conditions and incubated for 24 h at 37 °C.

### 2.8 Histomorphology of intestine

Histomorphological examination involved obtaining a 2 cm sample from the middle portion of the duodenum. Four chickens per group were randomly selected from the six sampled chickens ( n=4 ) after complete resection of the small intestine. The detailed procedure for this examination was conducted as previously outlined by Jahejo et al. (27). The tissue sections were examined and measured using a Nikon phase contrast microscope coupled with a microcomputer-integrated digital imaging analysis system (Nikon Eclipse 80i, Nikon Co., Tokyo, Japan).

### 2.9 Histomorphology of liver

Liver tissue samples were collected on day 35 and fixed in 10% neutral buffered formalin. Following fixation, tissues were processed using standard histological protocols: samples were dehydrated through a graded ethanol series, cleared in xylene, and embedded in paraffin wax. Sections of 5 μm thickness were cut using a microtome (Leica Microsystems, Wetzlar, Germany), mounted on glass slides, and stained with hematoxylin and eosin (H&E). Histopathological changes were examined using a Nikon phase contrast microscope (Nikon Eclipse 80i, Nikon Co., Tokyo, Japan). While a formal numerical scoring system for air sac and pericardial lesions was not employed, systemic disease severity was assessed through detailed gross pathological observations and high-resolution histopathological examination of the liver and intestines.

### 2.10 Bioinformatics analysis and target selection

To identify the most relevant immune markers for our study, we first conducted an exploratory bioinformatic screen using a public MDV-infected erythrocyte dataset. This allowed us to identify a core set of interleukin genes that are strongly modulated in avian erythrocytes, which we then validated in our *E. coli*-challenge model. We utilized the dataset GPL8764 (Agilent-015068 Chicken Gene Expression Microarray 4x44k), specifically comparing gene expression profiles between MDV-infected (GSM738766-GSM738769) and Control (GSM738774-GSM738777) subjects. Data were imported into R software using the GEO query package, and differentially expressed genes (DEGs) were identified using the *limma* package. Genes with an adjusted P<0.05 were considered significant. Gene Ontology (GO) and KEGG pathway enrichment analyses were performed to select key cytokine genes involved in immune response pathways for validation in the present study.

### 2.11 Erythrocyte isolation, RNA extraction, and quantitative real-time PCR (qRT-PCR)

To evaluate the specific immune response of erythrocytes, cells were purified from blood samples collected on day 21 ( n=3 per group) following the protocol described by St. Paul et al. (16). Briefly, blood samples were collected into tubes containing anticoagulant and diluted 1:1 with PBS. The diluted blood was layered onto a Histopaque-1119 density gradient (Sigma-Aldrich, St. Louis, MO, USA) and centrifuged at 400 × g for 20 min. The supernatant and buffy coat (containing leukocytes and thrombocytes) were discarded, and the erythrocyte pellet was harvested from the bottom layer. To remove potential contamination by adherent cells (such as monocytes), the erythrocytes were washed three times in RPMI-1640 medium (Gibco, Thermo Fisher Scientific) and subsequently incubated in T75 tissue culture flasks for 1.5 h at 41 °C. The non-adherent erythrocytes were then collected. The purity of the erythrocyte preparation was assessed by preparing blood smears and using Wright–Giemsa staining. Purity was quantified by counting cells in 10 random fields of view under oil immersion microscopy (100x magnification), calculating the ratio of erythrocytes to the total number of cells counted. The purity was determined to be >99% .

Total RNA was extracted from the purified erythrocytes using RNAiso Plus (Takara Bio Inc., Dalian, China) following the manufacturer’s protocol. RNA concentration and purity were assessed using a NanoDrop ND-1000 spectrophotometer (Thermo Scientific, Wilmington, DE, USA). First-strand cDNA was synthesized from 1 μg of total RNA using the PrimeScript™ RT Reagent Kit with gDNA Eraser (Takara Bio Inc., Dalian, China). Quantitative Real-Time PCR was performed using the TB Green® Premix Ex Taq™ (Takara Bio Inc.) on an Applied Biosystems 7,500 Real-Time PCR System. The reaction mixture (20 μL) contained 10 μL of SYBR Green Premix, 0.4 μL of each forward and reverse primer (10 μM) (Table 3), 2 μL of cDNA template, and 7.2 μL of nuclease-free water. The thermal cycling conditions were: 95 °C for 30 s, followed by 40 cycles of 95 °C for 5 s and 60 °C for 34 s. The relative gene expression levels were calculated using the $2^{-\text{ΔΔCt}}$ method, with *18S rRNA* serving as the internal reference gene.

**Table 3:** Primer sequences used in qPCR.

Genes	Primer (5′ → 3′)	Gene bank accession number
Interleukin-2 (IL-2)	F-CCCGTGGCTAACTAATCTGCTG	HE608819
	R-TGAGACACCAGTGGGAAACAGT	
Interleukin-6 (IL-6)	F-GAAATCCCTCCTCGCCAATCTGA	JN639847
	R-TGAAACGGAACAACACTGCCATCT	
Interleukin-10 (IL-10)	F-CTGAAGGCGACGATGC	EF554720
	R-TTCCTCCTCCTCATCAGC	
Interleukin-12 (IL-12)	F-GCCGACTGAGATGTTCCTGG	JN942590
	R-CCTTGCTTTTGTATTTCTTTGTGC	
Housekeeping gene (18S rRNA)	F: TTCCGATAACGAACGACAC	FM165414
	R: GACATCTAAGGGCATCACAG	

### 2.12 Statistical analysis

Data were analyzed using the General Linear Model (GLM) procedure of SAS software (Version 9.2; SAS Institute Inc., 2008). The analysis was validated to ensure mathematical consistency and reproducibility of the results. The pen served as the experimental unit. Fisher’s protected least significant difference (LSD) test was used to compare treatment means. Results are expressed as mean ± standard error of the mean (SEM). Statistical significance was set at P<0.05 .

## 3 Results

### 3.1 Growth performance & relative weight of organs

The success of the *E. coli* challenge was confirmed by the observation of clinical signs of colibacillosis and a significant increase in bacterial loads in the blood and cecum of the Infected group. Clinical observations revealed that chickens in the Infected group exhibited signs of depression, ruffled feathers, and mild diarrhea post-challenge, which were less pronounced in the camel milk supplemented groups (T1 and T2); however, the overall mortality rate was low (<5%) and showed no significant difference among the treatment groups. In broiler chickens challenged with *E. coli,* there was no significant difference in feed intake (FI) between the Control and Infected groups ( P>0.05 ), although the Control group demonstrated significantly (*p* < 0.05) higher weight gain (WG). When comparing the camel milk-supplemented groups, the T1 group (5 g/kg) exhibited significantly (*p* < 0.05) superior FI and WG compared to the T2 group (10 g/kg). When comparing the camel milk-supplemented groups, the T1 group (5 g/kg camel milk) exhibited significantly (*p* < 0.05) superior FI and WG compared to the T2 group (10 g/kg camel milk supplementation). Furthermore, the T1 group also showed a significantly (*p* < 0.05) more favorable feed conversion ratio (FCR) when compared to both the T2 group and the unsupplemented Infected group. These findings collectively suggest that a 5 g/kg camel milk supplementation yielded better overall growth performance outcomes in *E. coli*-challenged broiler chickens than a 10 g/kg supplementation level. Conversely, water intake was significantly higher in the T2 group compared to the T1 group (Figure 1A).

**Figure 1:**
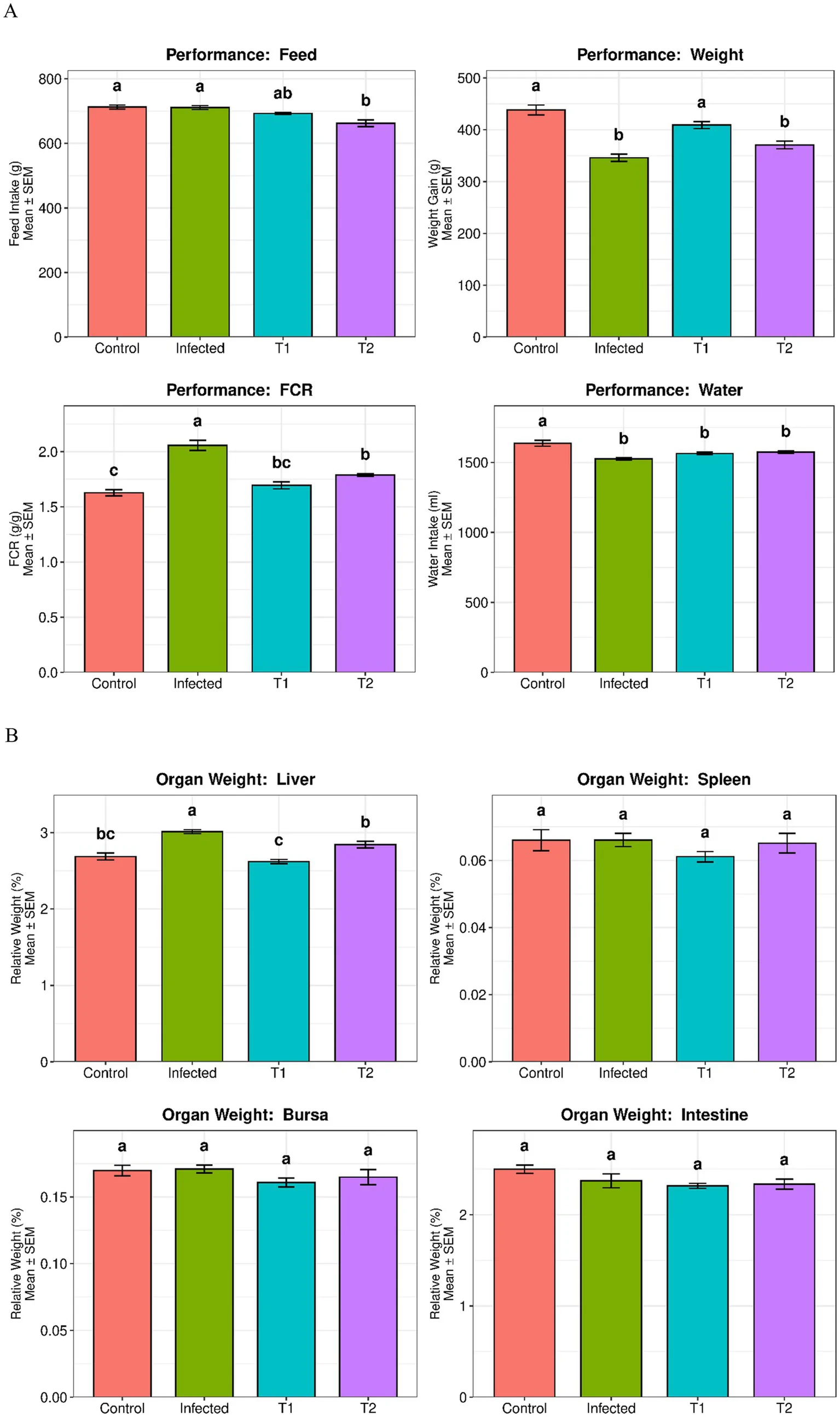
Effect of camel milk supplementation on growth performance and organ indices in *E. coli*-challenged broiler chickens. **(A)** Growth performance metrics including feed intake (g), weight gain (g), and feed conversion ratio (FCR; g feed/g gain) measured over the 35-day experimental period. Data represent the Mean ± SEM of 3 replicates per group (20 chickens/replicate). **(B)** Relative weights of lymphoid organs (Spleen, Bursa) and liver collected on day 35, expressed as a percentage of live body weight (%). Data represent the Mean ± SEM of 6 chickens per group (*n* = 6). Different letters (a, b, c) indicate significant differences (*p* < 0.05) among groups. Groups: Control (Basal diet + PBS), Infected (Basal diet + *E. coli*), T1 (*E. coli* + 5 g/kg Camel Milk), T2 (*E. coli* + 10 g/kg Camel Milk).

Assessment of relative organ weights in *E. coli*-challenged broiler chickens supplemented with camel milk powder revealed distinct patterns. The T2 group (10 g/kg camel milk) exhibited a significantly higher relative liver weight compared to the Control and T1 groups ( P<0.05 ), although it remained significantly lower than that of the Infected group. In contrast, the relative weights of the spleen, bursa, and intestine showed no significant differences across the experimental groups.

Camel milk supplementation notably influenced the microbial profile. Specifically, the T1 group (5 g/kg camel milk) displayed a significant reduction (*p* < 0.05) in the counts of most microflora compared to other treated groups (Figure 1B).

### 3.2 Hematology and viable bacteria (*Escherichia coli*) count in the blood and intestine

Analysis of hematological parameters on day 35 revealed significant ( P<0.05 ) differences across the experimental groups (Figure 2A). Hemoglobin (Hb) levels and packed cell volume (PCV) were significantly lower in the Infected group but were significantly increased in the T2 group compared to the Infected group, suggesting a recovery from infection-induced anemia. Conversely, red blood cell (RBC) and white blood cell (WBC) counts were significantly elevated in the Infected group; however, supplementation with camel milk, particularly in the T2 group, significantly reduced these counts toward the levels observed in the Control group, indicating a restoration of hematological homeostasis and a reduction in the systemic inflammatory response.

**Figure 2:**
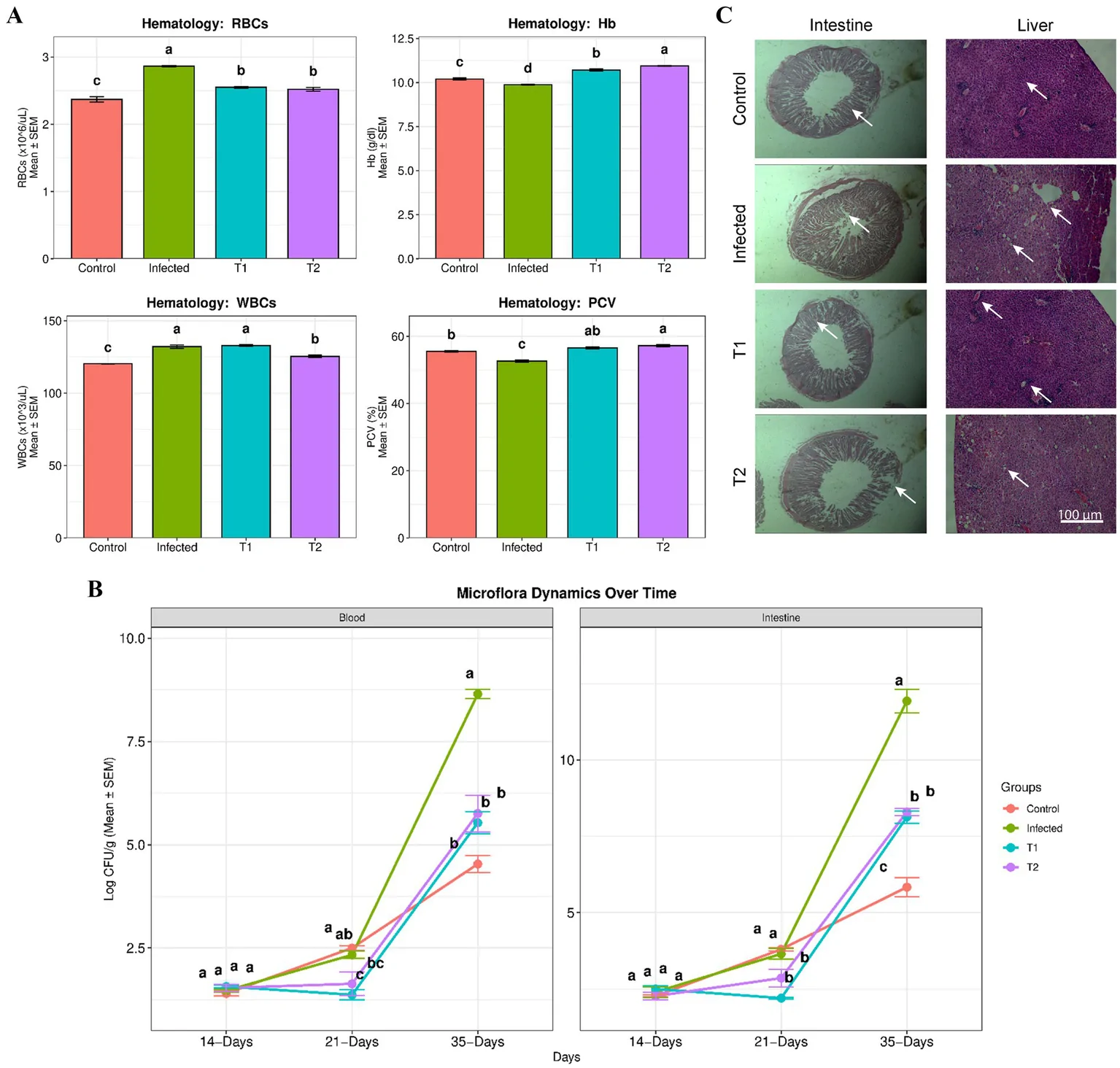
Hematological parameters, bacterial loads, and histomorphology in *E. coli*-challenged broilers. **(A)** Hematological indices (RBC, WBC, Hb, PCV) assessed on day 35 ( n=6 chicken/group). **(B)** Viable *E. coli* counts (CFU) in blood and cecal digesta measured on days 14, 21, and 35 ( n=6 chickens/group). *E. coli* was not detected in the blood samples of the Control group. **(C)** Representative photomicrographs of duodenum and liver histomorphology stained with H&E (*n* = 4 chickens/group). White arrows indicate characteristic pathological lesions (such as hepatocellular necrosis and mucosal damage) in the Infected group, and the relative preservation of tissue architecture in the T1 and T2 groups. Scale bar = 100 μ m. Data in bar charts represent Mean ± SEM. Different letters indicate significant differences ( P<0.05 ) among groups: Control, Infected, T1 (5 g/kg Camel Milk), and T2 (10 g/kg Camel Milk).

The enumeration of viable *E. coli* cells in the blood showed dynamic changes over the experimental period. No viable *E. coli* were detected in the blood of the Control group throughout the experiment. On day 14, bacterial counts in the blood did not differ significantly among the groups. However, by day 21, the Infected group displayed significantly ( P<0.05 ) higher viable *E. coli* counts, followed by the T1 and T2 groups. This trend persisted until day 35, where the Infected group maintained the highest blood *E. coli* counts ( P<0.05 ), while the T2 group exhibited the lowest counts among the challenged groups, followed by T1 (Figure 2B).

Similar to the blood profile, *E. coli* counts in the cecal contents varied over time. On day 14, no significant difference were observed among the groups. By day 21, the Infected group exhibited significantly ( P<0.05 ) higher cecal *E. coli* counts compared to the Control. On day 35, the Infected group maintained the highest bacterial load ( P<0.05 ). However, camel milk supplementation significantly reduced this load, with the T2 group showing a greater reduction in cecal *E. coli* counts compared to the T1 group (Figure 2B).

### 3.3 Intestinal and liver histomorphology

The histopathology of the intestine (duodenal) in camel milk supplementation broiler chickens was observed using the hematoxylin and eosin staining (Figure 2C). The control group of broiler chickens seems a moderate number of duodenal villi comparatively. The blood vessels in the Infected group were observed damaged in the lamina propria with similar length to Control group. However, the villi in group T2 appeared longer than those in the Infected group, although they were shorter than those observed in the T1 group. Overall, a more preserved villus structure was qualitatively observed in the camel milk-supplemented groups.

The histopathological examination of liver tissues from the camel milk supplementation group was conducted using hematoxylin and eosin (H&E) staining. In the Control group, hepatocytes appeared normal, exhibiting a regular shape, tightly packed arrangement, and no signs of necrosis. In contrast, the Infected group showed clear evidence of hepatocellular necrosis, with disorganized cell structures. The T2 group, which received camel milk supplementation, displayed hepatocytes with a less dense arrangement compared to the Control group, yet the overall hepatic architecture was relatively preserved and appeared qualitatively more organized compared to the Infected group. (Figure 2C).

### 3.4 Identification of DEGs in infected and control chicken erythrocyte

To identify candidate immune genes for validation in our experimental model, we first analyzed the public MDV-infected erythrocyte dataset. We examined a total of four biological replicates from both the Control group (accession numbers GSM738774, GSM738775, GSM738776, GSM738777) and the MDV-infected group (accession numbers GSM738766, GSM738767, GSM738768, GSM738769). Our differential gene expression analysis identified a total of 990 differentially expressed genes (DEGs) when comparing the MDV-infected group to the Control. Within this set, 467 genes exhibited a significant downregulation, while 523 genes showed significant upregulation in the MDV-infected chickens relative to the Control (Figures 3A,B).

**Figure 3:**
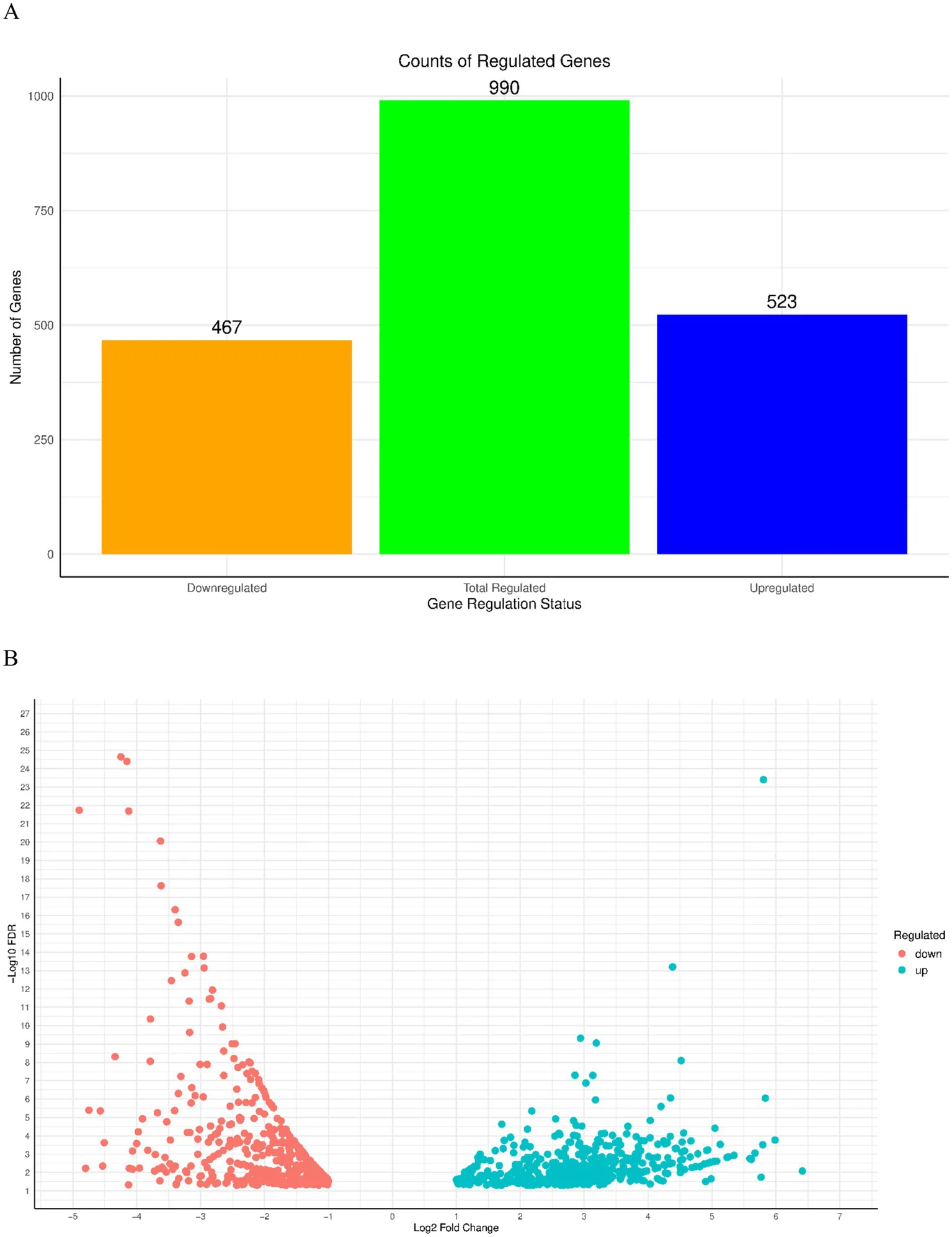
Differential gene expression analysis of erythrocytes from MDV-infected vs. Control chickens. **(A)** Bar plot illustrating the total count of significantly upregulated and downregulated genes. **(B)** Scatter plot (Volcano plot) displaying the distribution of differentially expressed genes (DEGs) based on log2 fold change and significance. Data were derived from the microarray dataset GPL8764 using 4 biological replicates per group ( n=4 ). Significance was set at adjusted P<0.05 .

### 3.5 GO functional enrichment analysis

Gene Ontology (GO) functional enrichment analysis categorized the identified differentially expressed genes (DEGs) into three main classifications: biological process (BP), cellular component (CC), and molecular function (MF) (Figure 3).

Among the upregulated DEGs, several key biological processes were significantly enriched. These included ‘Positive Regulation of Apoptotic Process,’ ‘Negative Regulation of Transcription from RNA Polymerase II Promoter,’ ‘Signal Transduction,’ ‘Single-organism Developmental Process,’ and ‘Axon Guidance.’ Additional enriched biological processes were ‘Positive Regulation of Transcription from RNA Polymerase II Promoter’ and ‘Negative Regulation of Apoptotic Process.’

For cellular components, the upregulated DEGs predominantly enriched terms such as ‘Membrane,’ ‘Nucleolus,’ ‘Integral Component of Membrane,’ ‘Plasma Membrane,’ ‘Cytoplasm,’ ‘Nucleus,’ and ‘Cytosol.’

Regarding molecular functions, the upregulated DEGs showed enrichment in categories including ‘Actin Binding,’ ‘Calcium Ion Binding,’ ‘Protein Kinase Binding,’ ‘Zinc Ion Binding,’ ‘Protein Homodimerization Activity,’ ‘ATP Binding,’ ‘Metal Ion Binding,’ and ‘Protein Binding’ (Figure 4A).

**Figure 4:**
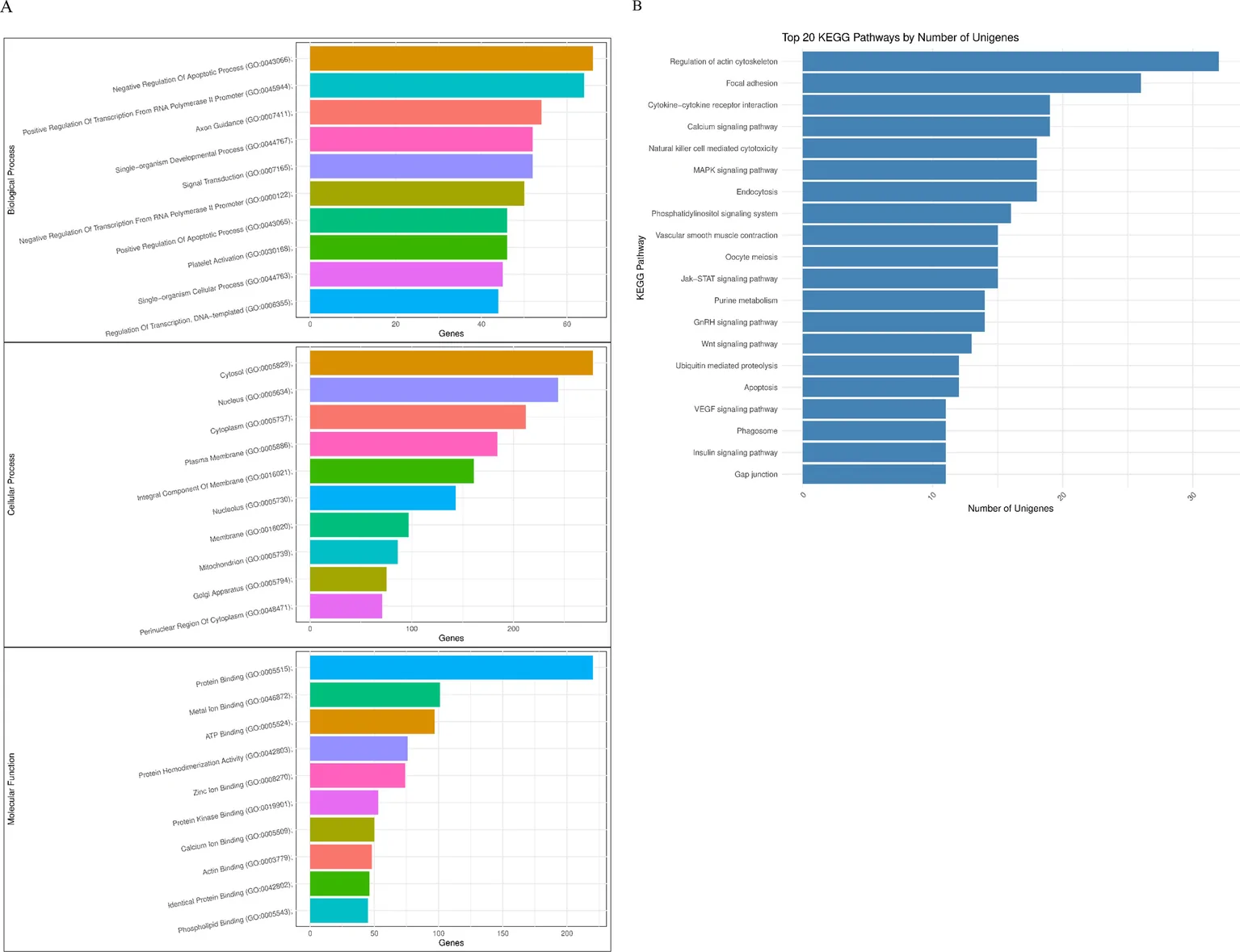
Functional enrichment analysis of DEGs identified in chicken erythrocytes. **(A)** Gene Ontology (GO) classification categorizing DEGs into Biological Process (BP), Cellular Component (CC), and Molecular Function (MF). **(B)** KEGG pathway enrichment analysis showing the top signaling pathways associated with the upregulated genes. Analysis is based on n=4 biological replicates per group.

### 3.6 Signaling pathway analysis in mRNA sequencing

Following pathway enrichment analysis, our findings indicate that upregulated genes were primarily associated with several key signaling cascades and metabolic processes. These significantly enriched pathways included ‘Apoptosis,’ ‘Ubiquitin-mediated proteolysis,’ ‘Wnt signaling pathway,’ ‘GnRH signaling pathway,’ ‘Purine metabolism,’ ‘Jak–STAT signaling pathway,’ ‘Oocyte meiosis,’ and ‘Vascular smooth muscle contraction.’ Additionally, pathways such as ‘Phosphatidylinositol signaling system,’ ‘Endocytosis,’ ‘MAPK signaling pathway,’ ‘Natural killer cell mediated cytotoxicity,’ and ‘Regulation of actin cytoskeleton’ were also prominently enriched (Figure 4B).

### 3.7 Identification of enriched immunity-related genes and signaling pathways

The heatmap and scatter plot shows the most enriched genes found in the Control and Infected chicken group are IL1RAP; IL15RA; BMPR2; MPL; PDGFB; CSF2RB; IL17RA; IL7R; IFNAR2; IFNAR1; TNFRSF21; IL10RA; IL1R1; PRH1; TNFRSF25; CCR7; IKBKE.

CD80; IFNAR2; IFNAR1; PIK3R5; PSTPIP1; XIAP; MAPK12; LCK; VAV1; PRKCB; VAV1 (Figures 5A,B). The enriched pathways are B cell receptor signaling pathway; T cell receptor signaling pathway; NOD−like receptor signaling pathway; Toll−like receptor signaling pathway; Cytokine−cytokine receptor interaction (Figure 5B).

**Figure 5:**
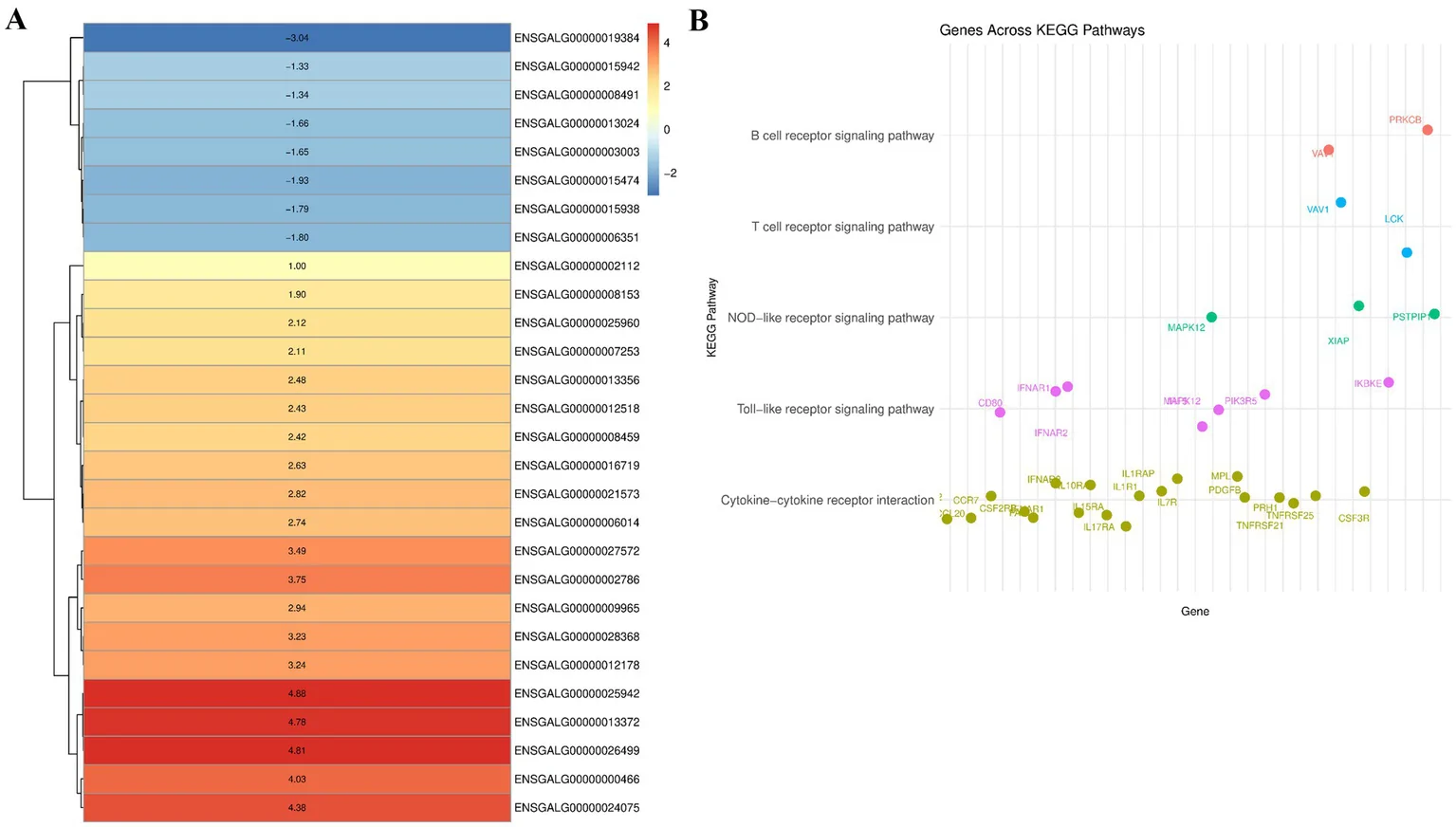
Immune-related gene signatures and pathway enrichment. **(A)** Heatmap clustering of the most significantly enriched immunity-related genes in Control vs. MDV-infected groups. **(B)** Scatter plot emphasizing key immune signaling pathways (B cell receptor, T cell receptor, NOD-like receptor, Toll-like receptor signaling) and associated gene distribution. ( n=4 biological replicates per group).

### 3.8 Validation of immunity-related genes on qRT-PCR

Based on above enriched signaling pathways and their immunity-related interleukin gene expression was significantly influenced by camel milk supplementation (Figure 6). For IL-2, expression was significantly higher in the Infected group compared to both the T1 and T2 groups ( P<0.05 ). Similarly, IL-12 expression was significantly downregulated in T1 and T2 groups compared to the Infected group ( P<0.05 ). For IL-6 and IL-10, the Infected group exhibited the highest expression levels, which were significantly reduced in the T2 group and further reduced in the T1 group ( P<0.05 ). Notably, the expression levels of IL-6 and IL-10 in the T1 group were restored to levels comparable to the Control group, indicating a successful mitigation of the inflammatory response.

**Figure 6:**
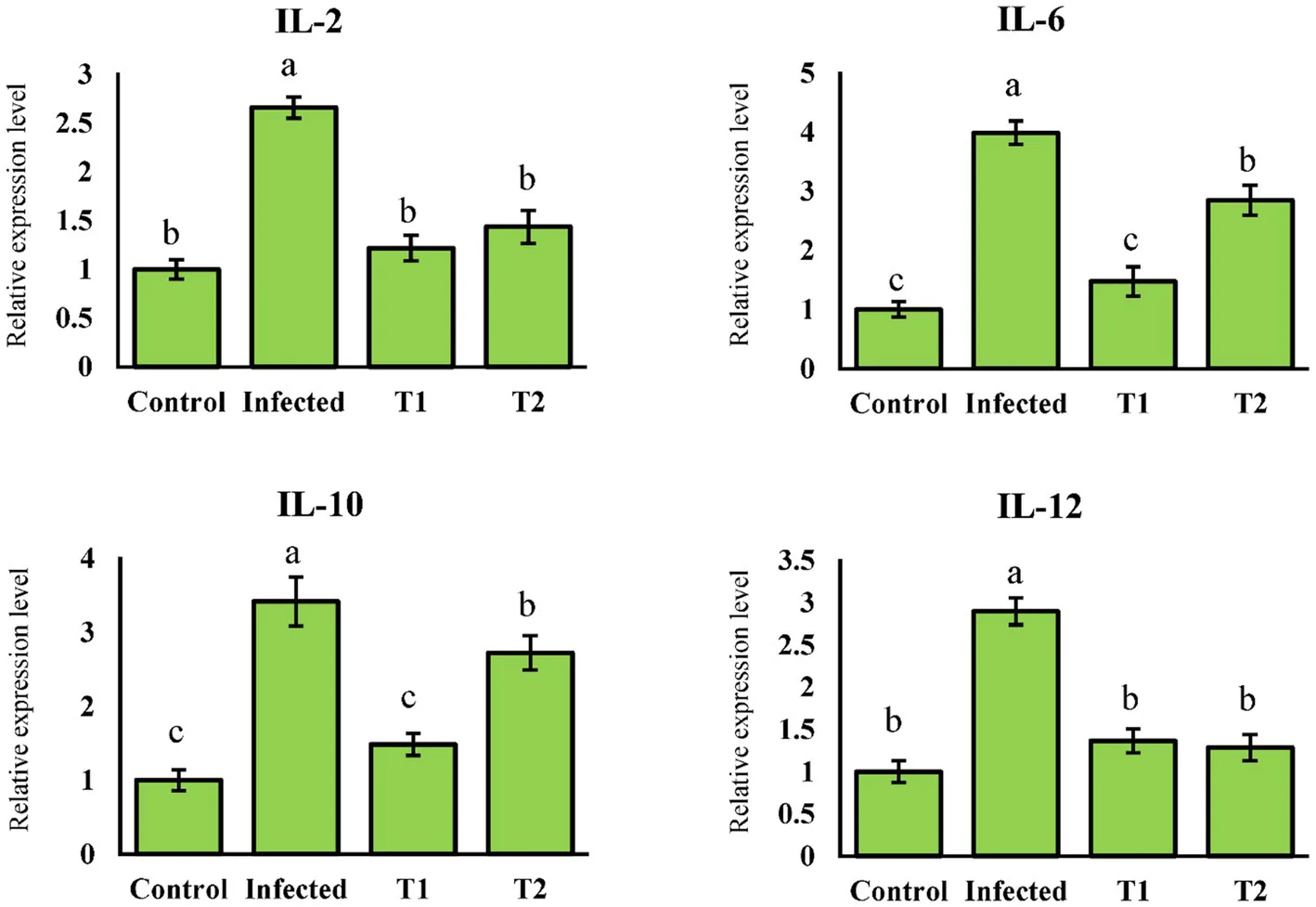
Validation of immunity-related interleukin gene expression in purified erythrocytes. Relative mRNA expression levels of Interleukin-2 (IL-2), IL-6, IL-10, and IL-12 normalized to the internal reference gene *18S rRNA*. Data represent the Mean ± SEM of 3 biological replicates per group ( n=3 ). Different letters (a, b, c) indicate statistically significant differences ( P<0.05 ) between groups. Groups: Control (Basal diet), Infected (*E. coli* challenged), T1 (*E. coli* + 5 g/kg Camel Milk), T2 (*E. coli* + 10 g/kg Camel Milk).

## 4 Discussion

The emergence of antibiotic-resistant *Escherichia coli* (*E. coli*) strains and the subsequent restrictions on antibiotic use in poultry feed have necessitated the search for safe, effective alternatives (28, 29). This study investigated the potential of camel milk powder—a rich source of bioactive antimicrobial peptides (AMPs)—to mitigate the detrimental effects of *E. coli* infection. To our knowledge, this is the first study to evaluate the regulatory role of camel milk supplementation on erythrocyte-related immune gene expression in *E. coli*-challenged broiler chickens.

In the present study, while the *E. coli* challenge did not significantly affect feed intake, it significantly depressed weight gain compared to the Control group. This suggests that the reduction in growth was not due to a lack of appetite (reduced FI), but rather to the systemic impact of the infection on nutrient utilization and metabolic efficiency. Interestingly, the T2 group (10 g/kg) showed a reduction in feed intake compared to T1, likely due to a dose-dependent effect on palatability or nutrient density. This further reinforces that 5 g/kg is the optimal inclusion level to improve performance without depressing feed consumption, a finding consistent with established models of avian colibacillosis (30). However, supplementation with 5 g/kg camel milk (T1) significantly ameliorated these growth deficits, improving the Feed Conversion Ratio (FCR) to levels comparable with the Control. These results align with previous findings by Daneshmand et al. (31), who reported that camel lactoferrin (cLF36) acts as an effective AMP, enhancing performance and intestinal morphology in challenged broilers. The improved growth performance in the T1 group can be directly attributed to the improved gut health and nutrient absorption resulting from the significant reduction in bacterial load observed in the cecum and blood (Figure 2B). The observation of reduced E . coli loads in the cecum, despite the intranasal challenge, suggests that camel milk supplementation may enhance the intestinal barrier or modulate the systemic immune response, thereby limiting the secondary colonization of the gut during systemic colibacillosis.

The reduction in viable *E. coli* counts in the blood and intestine of camel milk-supplemented birds (T1 and T2) highlights the potent antimicrobial properties of the supplement. This effect is likely mediated by the bioactive components of camel milk, specifically lactoferrin, lysozyme, and lactoperoxidase (12). Lactoferrin exerts a bacteriostatic effect by chelating free iron, which is essential for bacterial proliferation, and a bactericidal effect by binding to the Lipid A portion of lipopolysaccharides (LPS) on Gram-negative bacteria, causing cell wall lysis (32, 33). Additionally, lactoperoxidase and lysozyme contribute to non-immune host defense by degrading bacterial cell walls (34). The histological improvements observed in the liver and duodenum of the T1 and T2 groups further support the hypothesis that camel milk creates a gut environment unfavorable for pathogen colonization, thereby preserving tissue integrity and function (35).

Beyond direct antimicrobial activity, this study explored the immunomodulatory role of camel milk by analyzing cytokine gene expression in erythrocytes. While erythrocytes have traditionally been viewed solely as oxygen carriers, recent evidence confirms their role in the avian immune response, expressing TLRs and responding to pathogens (16, 20). In this study, the Infected group exhibited elevated expression of pro-inflammatory cytokines, indicative of an acute immune response to the bacterial challenge. Conversely, the T1 group showed significantly lower expression levels of *IL-2*, *IL-6*, *IL-10*, and *IL-12* compared to the Infected group. While the Infected group exhibited a hyper-inflammatory response, camel milk supplementation—particularly at 5 g/kg (T1)—effectively downregulated these cytokines, bringing their expression levels back to a range comparable to the Control group.

The downregulation of *IL-2* and *IL-12* in the supplemented groups should not be interpreted as immune suppression, but rather as a resolution of inflammation or a modulated response due to the lower bacterial burden. *IL-12* is critical for bridging innate and adaptive immunity and driving Th1 responses (39, 40, 41). The significantly lower *IL-12* levels in T1 and T2 suggest that the immune system was not as heavily taxed as in the Infected group, likely because the antimicrobial action of the camel milk had already reduced the pathogen load, thereby preventing the need for a hyper-inflammatory response. This “dampening” of the cytokine storm helps prevent immunopathology and reallocates energy from fighting infection back to growth (36). This mirrors findings where other prebiotic or AMP interventions modulated humoral and cellular immunity without causing overstimulation (37, 38).

A notable finding in this study was the dose-dependent response to camel milk supplementation, where the 5 g/kg dose (T1) significantly outperformed the 10 g/kg dose (T2) in terms of feed intake, weight gain, and FCR. The reduced growth performance in the T2 group appears to be primarily driven by a decrease in feed intake (Figure 1A). This reduction in intake could be attributed to several factors: first, higher concentrations of camel milk powder may have negatively affected the palatability of the diet; second, the increased inclusion of milk powder might have caused a ‘nutrient dilution’ effect, where the increase in one component inadvertently reduced the intake of other essential nutrients in the basal mash. This suggests that the protective effects of camel milk follow a non-linear dose–response curve, where 5 g/kg represents the optimal threshold for enhancing broiler health under E . coli challenge. Exceeding this optimal dose may provide diminishing returns or even counteract growth benefits by depressing appetite.

In conclusion, this study demonstrates that *E. coli* challenge significantly compromises the growth performance and immune status of broiler chickens. However, dietary supplementation with camel milk powder effectively mitigated these negative effects, although a clear dose–response effect was observed. The 5 g/kg inclusion level (T1) was identified as the optimal dose, significantly improving weight gain and feed conversion efficiency compared to both the Infected and the 10 g/kg (T2) groups. This indicates that while camel milk is a potent non-antibiotic additive, its inclusion rate must be carefully optimized to avoid reducing feed intake. Future research should focus on identifying the specific bioactive fractions of camel milk responsible for these immunomodulatory effects and validating these findings in larger-scale field trials. Additionally, exploring synergistic applications of camel milk with other phytogenics or probiotics may offer a robust, antibiotic-free strategy to control colibacillosis and reduce antimicrobial resistance in the poultry industry.

## Data Availability

This research utilized publicly accessible datasets. Gene expression profiles were obtained from the Gene Expression Omnibus (GEO) database (http://www.ncbi.nlm.nih.gov/geo), specifically comprising data from MDV-infected groups (GSM738766-GSM738769) and Control groups (GSM738774-GSM738777) originally deposited by other authors. The underlying platform for these datasets was GPL8764 (Agilent-015068 Chicken Gene Expression Microarray 4x44k).

## Supplementary material

SUPPLEMENTARY FILE 1 In total differentially expressed genes.

SUPPLEMENTARY FILE 2 Key pathways and related differentially expressed genes.
